# Aptamer-based surface-enhanced resonance Raman scattering assay on a paper fluidic platform for detection of cardiac troponin I

**DOI:** 10.1117/1.JBO.25.9.097001

**Published:** 2020-09-08

**Authors:** Dandan Tu, Allison Holderby, Gerard L. Coté

**Affiliations:** a Texas A&M University, Department of Biomedical Engineering, College Station, Texas, United States; b Texas A&M University, Department of Chemistry, College Station, Texas, United States; c Texas A&M Engineering Experiment Station Center for Remote Health Technologies and Systems, College Station, Texas, United States

**Keywords:** point-of-care testing, surface-enhanced Raman spectroscopy, myocardial infarction, aptamer sensor

## Abstract

**Significance:** Cardiac troponin I (cTnI) is a primary biomarker for diagnosis of myocardial infarction (MI). In contrast to central laboratory tests for cTnI, point-of-care (POC) testing has the advantage of providing results when the patient is first encountered, which helps high-risk patients to be treated more rapidly and low-risk patients to be released in a timely fashion. A paper fluidic platform is good for POC testing because the paper is abundant, low cost, and disposable. However, current cTnI assays on paper platforms use antibodies as the recognition element, which has limitations due to the high cost of production and antibody stability issues at the POC.

**Aim:** To develop an aptamer-based assay on a paper strip using surface-enhanced resonance Raman spectroscopy (SERRS) for detection of cTnI in the clinically relevant range at the POC.

**Approach:** Gold nanoparticles (AuNPs) were functionalized with a Raman reporter molecule, malachite green isothiocyanate. The functionalized AuNPs were encapsulated in a silica shell and provided a SERRS signal using a handheld Raman system with a 638-nm excitation wavelength. A primary aptamer and a secondary aptamer of cTnI were used in a sandwich assay format to bind the cTnI on a test line of a paper fluidic platform. By measuring the SERRS signal from the test line, the concentration of cTnI was quantitatively determined.

**Results:** The aptamer-based SERRS assay on a paper strip had a detection range of 0.016 to 0.1  ng/ml for cTnI, had good selectivity for cTnI compared to three other markers, had good stability over 10 days, and had good performance in the more complex serum sample matrix.

**Conclusions:** The aptamer-based SERRS assay on a paper strip has the potential to provide a sensitive, selective, stable, repeatable, and cost-effective platform for the detection of cTnI toward eventual use in diagnosis of MI at the POC.

## 1 Introduction

Myocardial infarction (MI) is a cardiovascular disease related to myocardial necrosis due to a reduction of blood supply to the heart.^1^ MI affects a large population, and the overall prevalence in the United States for MI is 3% for adults ≥20  years of age between 2011 and 2014.^2^ Moreover, the mortality rate from MI is a problem, and it is estimated that ∼14% of the people who experience an MI will die from it.^2^ Cardiac troponin I (cTnI) is a primary biomarker for diagnosis of MI,^3^^,^^4^ and the clinical cutoff of cTnI for diagnosis of MI is determined by the 99th percentile concentration.^5^ Specifically, it was reported that the range of clinically acceptable concentrations of the cTnI was 0.01 to 0.1  ng/ml.^6^^,^^7^ Compared with the conventional method of sending samples to a central lab, point-of-care (POC) testing for cTnI can help diagnosis of MI in the field,^8^^,^^9^ which allows high-risk patients to be treated more rapidly and low-risk patients to be released in a more timely fashion.^10^ This enables the health care provider to apply treatment rapidly, which requires a less invasive intervention procedure and also leads to much lower mortality.^11^^–^^13^ Thus, it is of great interest to develop a sensitive POC method for detection of cTnI in the clinically relevant range.

Recently, various POC testing systems for cTnI have been developed;^14^^–^^29^ however, these systems have limitations in cost effectiveness (especially in the cost of the disposable cartridges), poor sensitivity, or limited stability of the recognition element. Paper-based sensing systems have demonstrated potential as a POC platform to implement assays due to the merits of paper (e.g., abundance, low cost, and ease of disposability).^30^^,^^31^ Surface-enhanced Raman spectroscopy (SERS), a sensitive optical method that provides good performance for detection of trace analytes in a sample,^32^ has been used for developing assays for detection of cTnI.^17^^–^^21^ Paper-based SERS assays, in particular, have been developed for sensitive detection of cTnI.^15^^,^^16^^,^^22^^,^^23^ However, these assays use antibodies as the recognition element,^15^^,^^17^^–^^23^ which has limitations due to selection difficulties, high costs of production, and stability issues.^33^ Aptamers, on the other hand, can be used as the recognition element for low-cost POC testing due to their nontoxicity, thermal stability, tolerance to a range of pH levels, and long shelf life.^34^^,^^35^ Moreover, compared with SERS, surface-enhanced resonance Raman scattering (SERRS) can provide a 10- to 100-fold higher signal,^36^ which can enhance the transduction signal received from low quantities of an analyte. To synthesize a SERRS active particle, a Raman reporter molecule (RRM) that has the frequency of the electronic transition close to the frequency of the excitation light is needed.^37^ Malachite green isothiocyanate (MGITC), a molecule with electronic transitions near 638 nm,^38^ was thus chosen as a good RRM to provide the SERRS signal.

In this paper, an aptamer-based assay on paper platform using SERRS active particles for detection of cTnI was developed. The hydrodynamic size distribution and the zeta potential of synthesized particles were characterized, and characteristic peaks of the SERRS spectrum were demonstrated. The performance of the developed aptamer-based assay on a paper platform using SERRS active particles for the quantitative detection of cTnI was evaluated. The selectivity of the assay against interfering molecules, stability of the assay over 10 days, and the performance of the assay in human serum were also evaluated.

## 2 Materials and Methods

### 2.1 Combined System Configuration

**Fig. 1:**
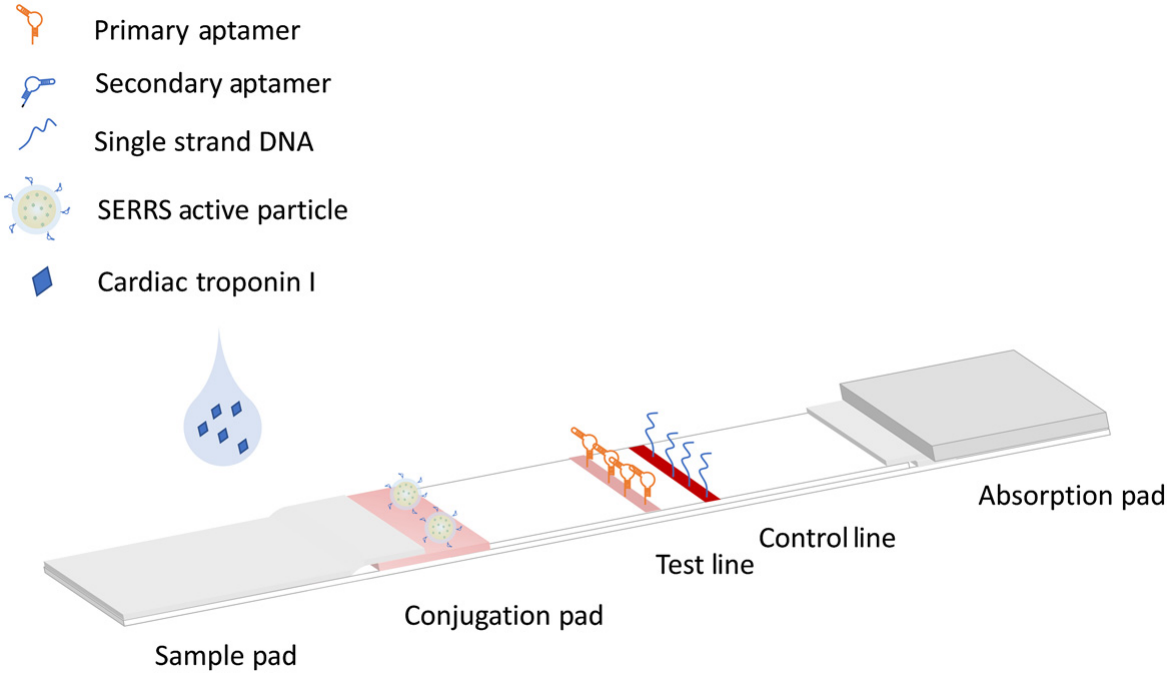
Schematic of the aptamer-based assay on a paper platform using SERRS active particles for detection of cTnI.

Configuration of the developed aptamer-based assay on a paper platform using SERRS active particles for detection of cTnI is shown in Fig. 1. SERRS active particles functionalized with a secondary aptamer of cTnI were stored in a conjugation pad. To measure a sample with cTnI, the sample was introduced onto the sample pad followed by the introduction of a running buffer. The sample resuspended the SERRS active particles and flowed to test line region. The cTnI formed a sandwich binding of the SERRS active particle, the cTnI protein, and the aptamer on the test line. Excess SERRS active particles flowed to the control line region and bound with DNA on the control line. In this configuration, the SERRS signal would increase with the amount of SERRS active particle bound on the test line, which is a direct result of an increase in the concentration of cTnI in the sample.

### 2.2 Materials and Instruments

cTnI was purchased from GenScript (Piscataway, New Jersey, USA). The sequence of aptamers for the cTnI assay was reported in previous journal papers.^39^^,^^40^ In this work, the primary aptamer of cTnI is 5′-biotin-TTTTTTCGTGCAGTACGCCAACCTTTCTCATGCGCTGCCCCTCTTA-3′. The secondary aptamer of cTnI is 5′-amine-spacer 18-spacer 18-CGCATGCCAAACGTTGCCTCATAGTTCCCTCCCCGTGTCC-3′. The sequence of the DNA strand on the control line is 5′-biotin-TTTTTTGGACACGGGGAGGGAACTATGAGGCAACGTTTGGCATGCG-3′. The DNA strands for cTnI assay were purchased from Integrated DNA Technologies (Iowa, USA). Whatman Grade 1 chromatography paper and Immunopore FP nitrocellulose paper were purchased from Cytiva (Formerly GE Healthcare Life Sciences, Marlborough, Massachusetts, USA). Glass fiber (GFCP000800) was purchased from EMD Millipore (Burlington, Massachusetts, USA). Thick blot filter paper was purchased from Bio-Rad (Hercules, California, USA). The mounting adhesive card was purchased from Michaels (Irving, Texas, USA). Milli-Q ultrapure water ($18.2\;M\Omega \;{\mathrm{cm}}^{-1}$) was used in all the procedures. (3-triethoxysilyl)propylsuccinic anhydride (TEPSA) was purchased from Gelest (Morrisville, Pennsylvania, USA). N-hydroxysulfosuccinimide sodium salt (sulfo-NHS) and N-ethyl-N′-(3-dimethylaminopropyl) carbodiimide hydrochloride (EDC) were purchased from CovaChem (Loves Park, Illinois, USA). MGITC was purchased from Thermo Fisher Scientific (Waltham, Massachusetts, USA), and methoxy-poly(ethylene glycol)-thiol (mPEG-SH, 10 kDa) was purchased from Nanocs (New York City, New York, USA). Human serum was purchased from Innovative Research (Novi, Michigan, USA). Other chemicals were purchased from Sigma-Aldrich (St. Louis, Missouri, USA).

Absorbance spectra of particles were measured on a Tecan Infinite 200 Pro (Tecan, Männedorf, Switzerland) microplate reader. Transmission electron microscopy (TEM) images were acquired on a JEOL JEM-2010 (JEOL, Tokyo, Japan). The zeta potential and hydrodynamic size of the particle were measured on a Zetasizer Nano ZS90 (Malvern, UK). All SERRS spectra were collected using an Ocean Optics portable Raman spectrometer (IDR-MINI) with a 638-nm laser (40-mW laser and 3-s exposure time). Three measurements were taken on one test line, moving from the left side to the right side, and the SERRS signal of the test line on this strip was the average of the intensity from these spectra. All spectra were baseline corrected.

### 2.3 Nanoparticle Synthesis

A seed-mediated synthesis method was used to synthesize the gold nanoparticles (AuNPs) with a size of around 60 nm.^41^ First, 75 ml of 2.2-mM sodium citrate in water was heated until boiled. Second, 0.5 ml of 25-mM chloroauric acid solution was injected. The mixture was stirred for 30 min, followed by adjusting the temperature to 90°C. Third, 0.5 ml of 25-mM chloroauric acid solution was injected, and the mixture was stirred for 30 min. This step was repeated one time, and then the particle solution was diluted by extracting 27.5 ml of solution and adding 26.5 ml of water and 1 ml of 60-mM sodium citrate. Fourth, 0.5 ml of 25-mM chloroauric acid solution was injected, and the mixture was stirred for 30 min. Steps two to four were repeated until the size of the particle reached the target size, and the particle solution was cooled down before using.

**Fig. 2:**
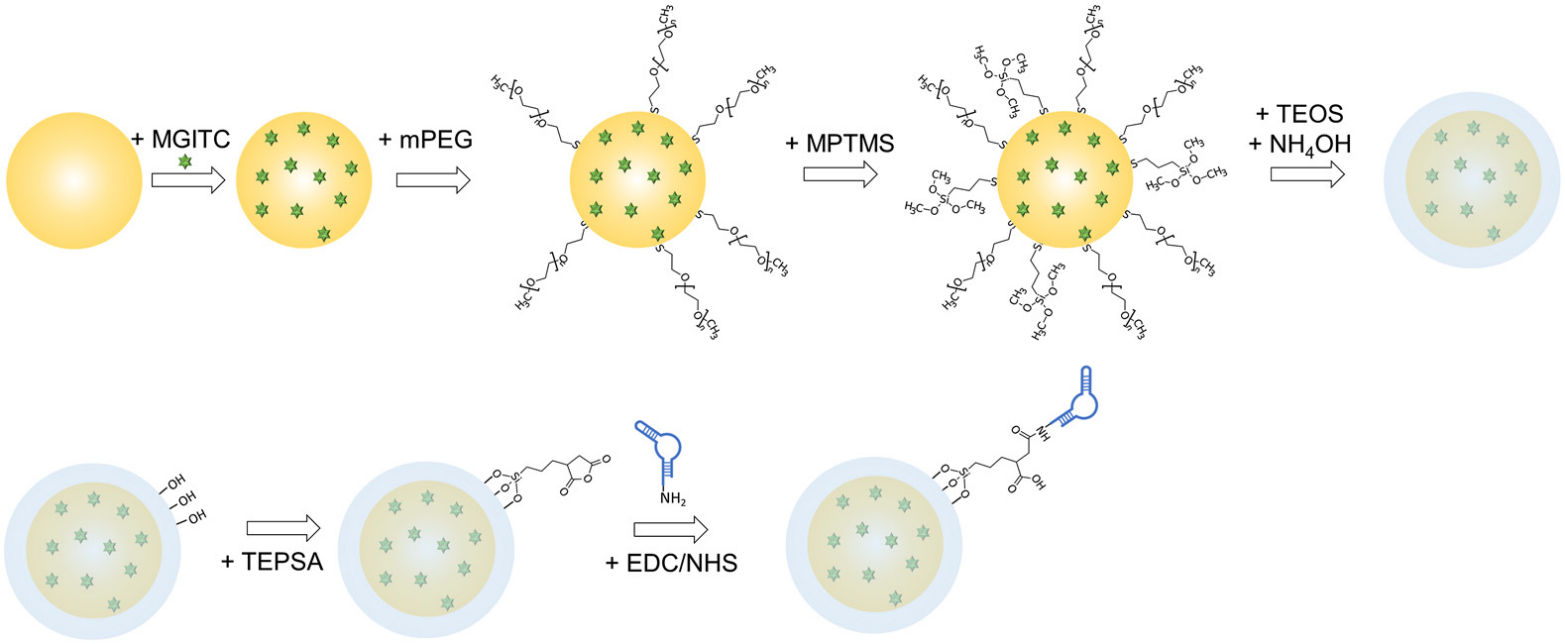
Process to synthesize the aptamer/silica/MGITC/AuNPs.

Following AuNPs synthesis, MGITC was functionalized on AuNPs as an RRM, and the MGITC-modified AuNP (MGITC/AuNP) was then encapsulated in a silica shell. The process is shown in Fig. 2. Specifically, 50  μl of 20-μM MGITC solution was mixed with 10 ml of AuNPs in a glass vial, and the mixture was kept shaking for 15 min. As a stabilizing component, 12.4  μl of 1-mM mPEG-SH was then added, and the mixture was kept shaking for 15 min. After centrifuging (1500 rcf, 20 min), the particle was resuspended in 1 ml of 2-propanol. To begin the silica shell formation, 3.6 ml of water and 19.7  μl of 0.1-mM (3-mercaptopropyl)trimethoxysilane (MPTMS) solution was added to the particle solution. After shaking for 15 min, 9 ml of 2-propanol, and 250  μl of ammonium hydroxide were added. Then, 100  μl of tetraethyl orthosilicate (TEOS) solution (1% in 2-propanol) was added and was kept rotating. After 30 min, another 100  μl of TEOS was added, and the mixture was kept rotating overnight. The resulting solution was centrifuged (1000 rcf, 20 min) and washed three times with ethanol. Finally, the synthesized silica shell MGITC-modified AuNP (silica/MGITC/AuNP) was resuspended in ethanol.

### 2.4 Nanoparticle Functionalization with Aptamer

As shown in Fig. 2, a secondary aptamer was covalently bound on the surface of the silica/MGITC/AuNP to form an aptamer-functionalized silica shell MGITC-modified AuNP (aptamer/silica/MGITC/AuNP). Before functionalization, the secondary aptamers were heated to 85°C for 5 min and slowly cooled to room temperature. Then, 1 ml of silica/MGITC/AuNP was mixed with 0.3 ml of water and 0.2 ml of 0.2-M TEPSA, followed by shaking overnight. The mixture was then centrifuged (1500 rcf, 15 min) and resuspended in 0.2 ml of phosphate buffer (pH 7.4) followed by mixing with 75  μl of 50-mM sulfo-NHS, 75  μl of 200-mM EDC, and 80  μl of 10-μM amine-functionalized aptamer. The mixture was sonicated and left shaking for 1 h followed by addition of 150  μl of 0.2-M ethanolamine (pH 8.6). The mixture was kept shaking for 1 h and the formed aptamer/silica/MGITC/AuNP was sonicated, centrifuged (1000 rcf, 15 min), and washed with phosphate buffer (pH 7.4).

### 2.5 Preparation of an Aptamer-Based Paper Strip

As shown in Fig. 1, the aptamer-based paper strip is composed of a sample pad, a conjugation pad, a nitrocellulose paper with a test line and a control line, and an absorption pad. The sample pad was prepared by cutting a Whatman Grade 1 chromatography paper into a strip with a width of 15 mm, soaking it in 0.05-M Tris buffer (pH 7.6, 0.15 M NaCl) with 0.25% Triton X-100, and drying it at 45°C for 1 h. The conjugation pad was prepared by soaking the glass fiber (8-mm width) in a buffer (1% bovine serum albumin, 0.5% Tween, and 5% sucrose), drying it at 45°C for 1 h, soaking the aptamer/silica/MGITC/AuNP solution, and drying it at 45°C for 1 h.

A primary aptamer of cTnI and a single-strand DNA, which was the reverse complimentary DNA strand of the secondary aptamer of cTnI, were immobilized on the nitrocellulose paper to form a test line and a control line, respectively. Biotin–streptavidin interaction was used to immobilize the aptamer and the single-strand DNA on the nitrocellulose paper. Before immobilization, the primary aptamers were heated to 85°C for 5 min and slowly cooled to room temperature. Then, 1 ml of 10-μM biotinylated primary aptamer was mixed with 40  μl of 2.5-mg/ml streptavidin, and the mixture was kept shaking for 1 h. The aptamers unbound to streptavidin were then washed by centrifugation (4300 rcf, 20 min) using a Nanosep (30 kDa). The produced primary aptamer/streptavidin conjugate was resuspended in 400  μl of phosphate-buffered saline (PBS). The same process was repeated to form the single-strand DNA/streptavidin conjugate. The nitrocellulose paper was cut into a strip with a width of 22 mm and was dispensed with the primary aptamer/streptavidin and the single-strand DNA/streptavidin to form the test line and control line, respectively. The nitrocellulose paper was dried at room temperature overnight. To block the remaining adsorption sites on the nitrocellulose paper, the nitrocellulose paper was immersed in 1% poly(vinyl alcohol) (PVA, molecular weight of 9000 to 10,000) aqueous solution for 30 min and dried at 45°C for 30 min.

The absorption pad is composed of a Whatman Grade 1 paper (5-mm width) and a thick blot filter paper (25-mm width). The Grade 1 paper is softer than the thick blot paper and it has the ability to wick fluid laterally. The thick blot filter paper can absorb large volumes of liquid. In this design, the Grade 1 paper was added to provide good contact between the nitrocellulose paper and the thick blot paper and ensure good wicking of waste solution. The sample pad, conjugation pad, functionalized nitrocellulose paper, and absorption pad were assembled on an adhesive card with 2 mm overlap between the pads. Finally, the assembled paper was cut into paper strips with a width of 4 mm and stored in a petri dish sealed with parafilm.

### 2.6 Response of the Assay

**Fig. 3:**
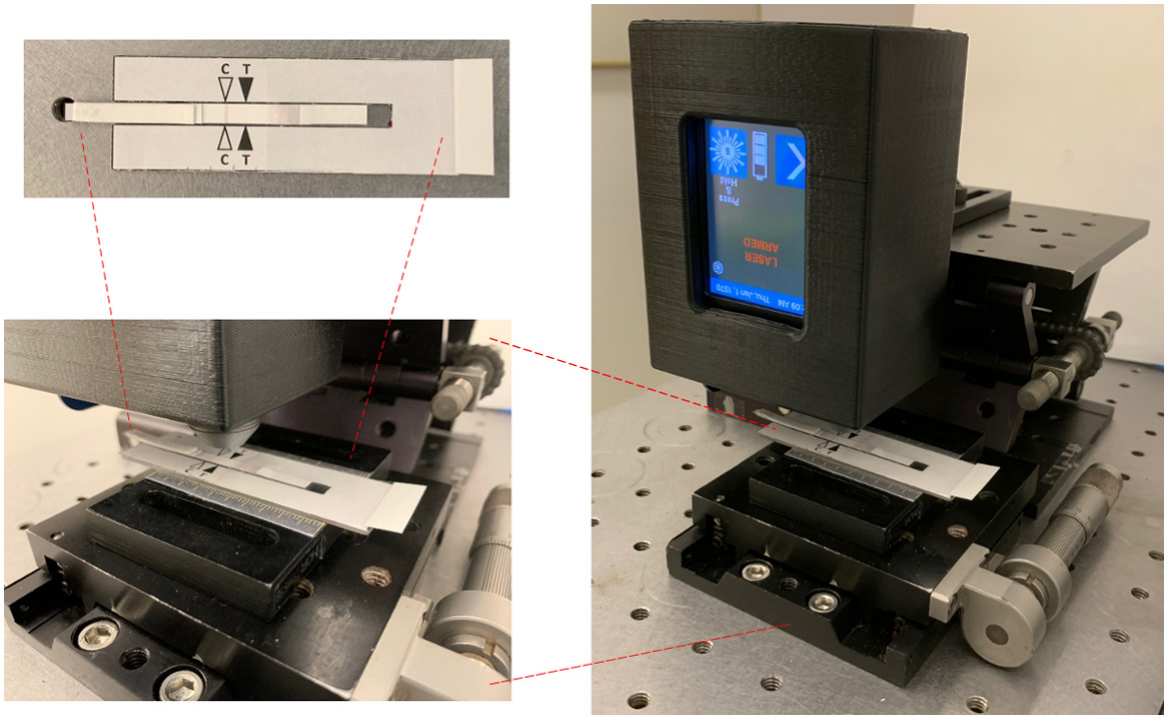
Setup of the portable Raman spectrometer to measure SERRS signal from a paper strip.

Different concentrations of cTnI (0, 0.01, 0.03, 0.05, and 0.1  ng/ml) in PBS (pH 7.4) were measured using the developed aptamer-based paper strip. To test a sample, 25  μl of the sample was dropped on the sample pad, and then 50  μl of running buffer (PBS with 0.25% Tween) was deposited on the sample pad after positioning the paper strip vertically. After all solution flowed to the end of the paper and was wicked by the absorption pad, a portable Raman spectrometer was used to measure the SERRS signal on the test line. To collect the SERRS signal, the paper strip was put in a strip holder (shown in Fig. 3). The control line was aligned with an indicator on the strip holder. After positioning the strip holder on the platform, SERRS signal was collected using the Raman spectrometer. Tests for each concentration were performed in triplicate.

### 2.7 Selectivity of the Assay

Selectivity of the aptamer-based paper strip was evaluated by comparing the SERRS signal to 0.1  ng/ml of cTnI and potential confounders, namely, c-reactive protein (CRP), heart-type fatty acid-binding protein (h-FABP), and B-type natriuretic peptide (BNP). The 25  μl sample of either 0.1  ng/ml of cTnI, CRP, h-FABP, or BNP was dropped on the sample pad, and then 50  μl of running buffer (PBS with 0.25% Tween) was applied on the sample pad after positioning the paper strip vertically. The paper strip wicked the samples, and the solution flowed down the paper and was wicked by the absorption pad. The portable Raman spectrometer was then used to measure the SERRS signal on the test lines. Tests for each sample were performed in triplicate. One-way analysis of variance was performed in OriginLab to determine whether the response to cTnI was significantly different from the response to these potential confounders.

### 2.8 Stability of the Assay

The paper strip was stored in a petri dish sealed with parafilm at room temperature. Stability of the aptamer-based paper strip was evaluated by recording the response of the paper strip to 0.03  ng/ml of cTnI over 10 days. Specifically, the response at day 1, day 2, day 4, day 7, and day 10 were collected. Tests for each day were performed in triplicate.

### 2.9 Application for the Analysis of the Serum Sample

The following test was implemented to evaluate the performance of the aptamer-based paper strip for detection of cTnI in a complex biological sample matrix. Without any pretreatment, human serum was spiked with cTnI to reach a concentration of 0.03 and 0.05  ng/ml of cTnI. The cTnI spiked serum samples were then used to test the developed aptamer-based paper strip. Specifically, 25 μl of sample was dropped on the sample pad, and then 50  μl of running buffer was applied on the sample pad after positioning the paper strip vertically. The paper strip wicked the samples and the solution flowed down the paper and was wicked by the absorption pad. The portable Raman spectrometer was then used to measure the SERRS signal on the test lines. Tests for each sample were performed in triplicate.

## 3 Result and Discussion

### 3.1 Characterization of Particle

**Fig. 4:**
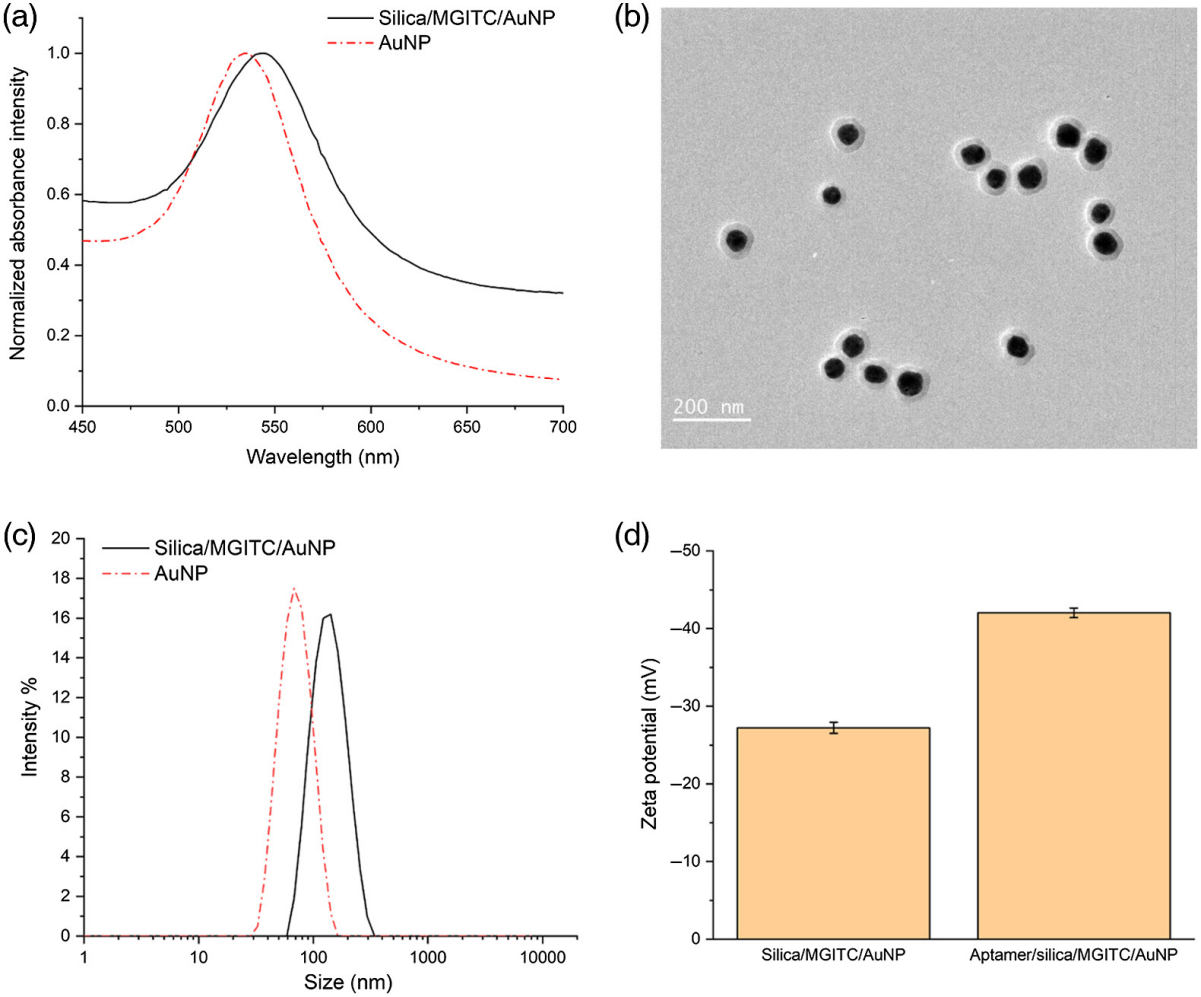
(a) Absorbance spectrum of synthesized AuNPs. (b) TEM image of silica/MGITC/AuNPs. (c) Size distribution of AuNPs and silica/MGITC/AuNPs. (d) Zeta potential of silica/MGITC/AuNPs before and after functionalization of the secondary aptamer.

Absorbance spectrum was used to confirm the synthesis and AuNPs. Figure 4(a) shows the absorbance spectrum of the synthesized AuNPs, in which the surface plasmon resonance peak was shown around 536 nm. The RRM MGITC, with electronic transitions near 638 nm,^38^ was used to provide the SERRS signal. Adsorption of MGITC reduced the electrostatic stability of the MGITC/AuNP particle, which could lead to aggregation of the particles during the processing steps, thus mPEG-SH was used to stabilize the particle. In addition, the binding force in Au–isothiocyanate interaction when adsorbing the MGITC to AuNP is weak, which can lead to fluctuations in the signal.^42^ Encapsulation of a silica shell was an efficient way to prevent reporter molecules from desorption from the particle surface.^43^ Thus, the MGITC/AuNP was encapsulated in a silica shell to produce a stable and reproducible SERRS signal. MPTMS was used to functionalize the surface of the MGITC/AuNP with an ethoxy group to facilitate the adsorption of TEOS.^44^ TEOS and ammonium hydroxide were added to form a silica shell. As shown in Fig. 4(a), there was a redshift of the absorbance peak of the synthesized silica/MGITC/AuNP compared with AuNPs, indicating the modification of MGITC and silica shell.

The overall morphology of synthesized silica/MGITC/AuNP was observed from the TEM image shown in Fig. 4(b). A clear silica shell encapsulating the Au core in the synthesized silica/MGITC/AuNP particles could be observed. It is also seen that almost all particles had an individual RRM-functionalized AuNP encapsulated in a silica shell, confirming the effect of mPEG-SH used to stabilize the particle and to prevent the particle from aggregating during the process used for particle encapsulation.

Figure 4(c) shows the hydrodynamic size distribution of the synthesized AuNPs and silica/MGITC/AuNP particles. As can be seen, the synthesized AuNPs have a mean size of 64 nm. Compared to a small-size AuNP, a large-size AuNP was used here to provide better efficiency for SERS with excitations at 630 to 650 nm.^38^ As shown in Fig. 4(c), after functionalizing MGITC and growing a silica shell, the size of the particle increased to around 129 nm. Moreover, the uniformity in size distribution of silica/MGITC/AuNP particles was close to that of the bare AuNPs, indicating the thickness of the formed silica shell was consistent.

To form an aptamer/silica/MGITC/AuNP, TEPSA was modified on the surface of the silica/MGITC/AuNP particle. The succinic anhydride group of TEPSA was hydrolyzed into two carboxyl groups, which was then activated by EDC and sulfo-NHS and reacted with the amine group of the secondary aptamer. After this process, the secondary aptamer was functionalized on the silica/MGITC/AuNP particle by an amide bond. Figure 4(d) shows the zeta potential change before and after functionalization of the secondary aptamer. After the functionalization, the zeta potential of the particles remained negative and had an increase in value, which was due to the strong negative charge of the aptamer.

**Fig. 5:**
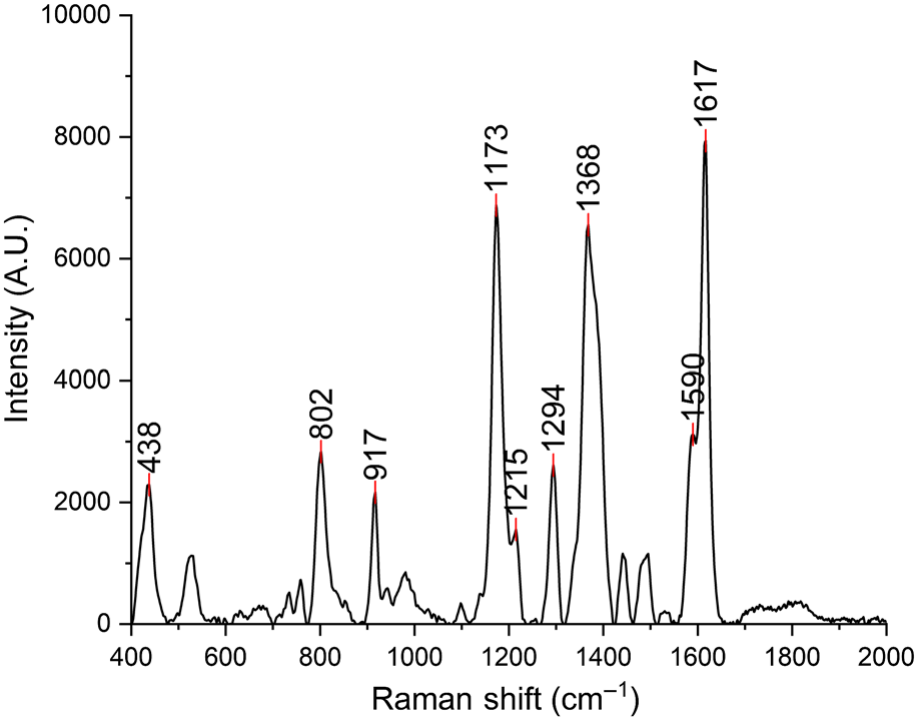
SERRS spectrum of the aptamer/silica/MGITC/AuNPs.

The SERRS spectrum of the synthesized aptamer/silica/MGITC/AuNP particle was measured and is shown in Fig. 5, and its characteristic peaks were from the vibrational modes of MGITC. The three strong characteristic peaks of the aptamer/silica/MGITC/AuNP particle were 1173, 1368, and $1617\;{\mathrm{cm}}^{-1}$.

### 3.2 Response of the Assay

The developed aptamer-based paper strip was used to measure different concentrations of cTnI. To measure a sample with cTnI, 25  μl of the sample was introduced onto the sample pad of the paper strip. The sample resuspended the aptamer/silica/MGITC/AuNPs, and the cTnI started to bind with the NPs. Then, 50  μl of the running buffer was introduced to drive the cTnI and the particle to flow to the end of the paper. The paper strip was positioned vertically when wicking the running buffer to achieve a low flow rate, which provided a longer reaction time for cTnI, the aptamer/silica/MGITC/AuNPs, and the primary aptamer on the test line. When cTnI was captured by the primary aptamer on the test line, a sandwich binding of the aptamer/silica/MGITC/AuNPs, the cTnI protein, and the primary aptamer was formed. The free aptamer/silica/MGITC/AuNPs not bound with cTnI passed the test line, flowed to the control line, and bound with the single-strand DNA on the control line. The more cTnI present in the solution, the more aptamer/silica/MGITC/AuNPs were captured on the test line, and thus there was a stronger SERRS signal. By measuring the SERRS signal, the concentration of target molecule was quantitatively determined. It took around 10 min for the sample to initially flow down the paper, and to increase the sensitivity by allowing more particles and sample to interact, it took around 40 min for the paper strip to wick both the sample and the running buffer to the absorption pad.

**Fig. 6:**
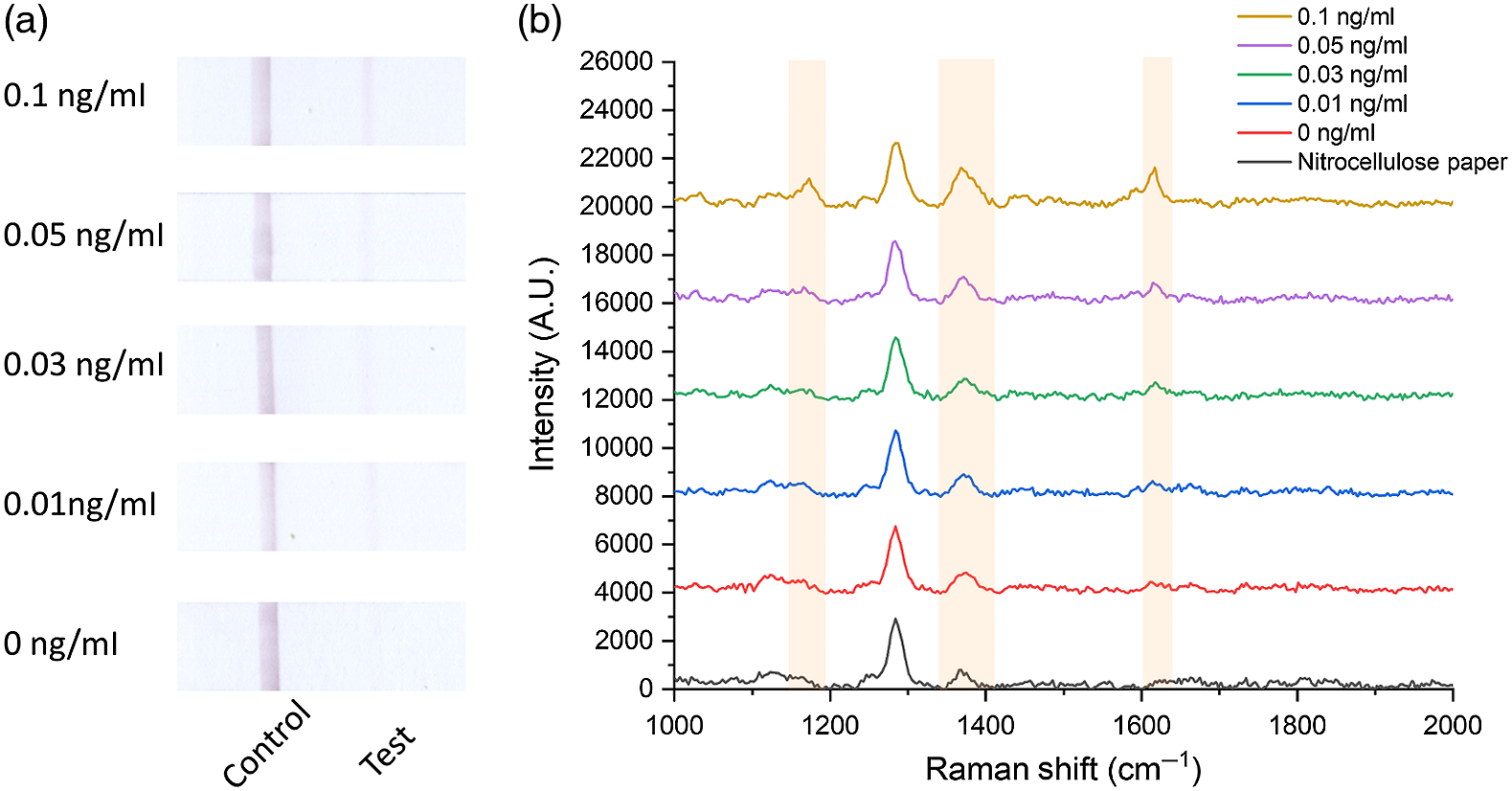
(a) Images of paper strips and (b) SERRS spectra from the test line on a blank nitrocellulose paper and the paper strips after loading different concentrations of cTnI.

Images of the paper strips after loading different concentrations of cTnI are shown in Fig. 6(a). It can be seen that the control line was easy to see with one’s bare eyes. The test line was not as strong or as easy to read as the control line, especially at the lower concentrations. However, using a portable Raman spectrometer to measure the signal from the test line, quantitative results could be obtained. The distribution of the signal on the test line is shown in Fig. S1 in the Supplementary Material. The SERRS spectra of the test line of the paper strips are shown in Fig. 6(b). Among the three strong characteristic peaks (1173, 1368, and $1617\;{\mathrm{cm}}^{-1}$), $1617\;{\mathrm{cm}}^{-1}$ was chosen as the best peak due to the nitrocellulose paper having a low background at this peak. By comparing the SERRS intensity at $1617\;{\mathrm{cm}}^{-1}$ from the test lines, the signal was shown to increase with an increasing concentration of cTnI.

**Fig. 7:**
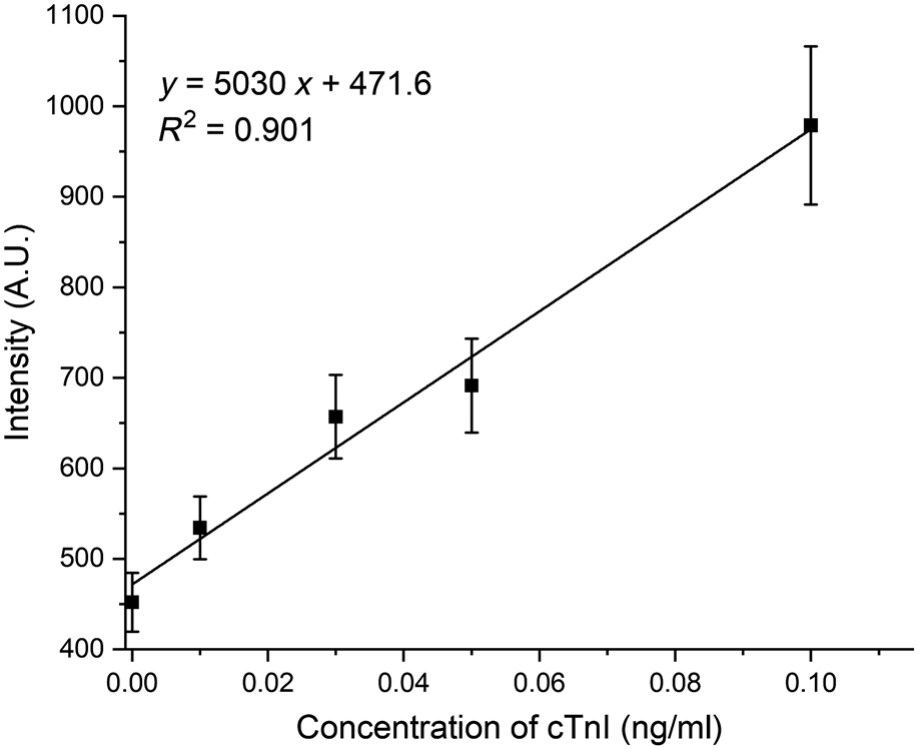
SERRS intensity at peak $1617\;{\mathrm{cm}}^{-1}$ of the test line of the aptamer-based paper strip to different concentrations of cTnI (n=3).

The correlation of the SERRS peak intensity at $1617\;{\mathrm{cm}}^{-1}$ with the cTnI concentration (0 to 0.1  ng/ml) is shown in Fig. 7. The SERRS signal observed for 0  ng/ml of cTnI could be due to some nonspecific binding of the secondary aptamer-functionalized silica particle with the primary aptamer/streptavidin on nitrocellulose paper. The results show that SERRS intensity was linearly correlated with the concentration of cTnI, and the limit of detection (LOD) was calculated to be 0.016  ng/ml. The dynamic range of the developed aptamer-based paper strip for cTnI was 0.016 to 0.1  ng/ml, which is close to the physiological relevant range of cTnI (0.01 to 0.1  ng/ml). This result indicates that the developed aptamer-based paper strip has a potential to be used to quantitatively detect cTnI at the POC. The comparison of the developed aptamer-based paper strip with previously reported assays for detection of cTnI is shown in Table S1 in the Supplementary Material. Compared with other paper-based assays,^16^^,^^25^^,^^26^ the developed paper strip used an aptamer, which is more thermally stable, has better tolerance to wide range of pH, and is a lower cost recognition element (>50 times lower cost compared to the cost of cTnl antibodies purchased online).^34^^,^^35^ In addition, using a portable reader, it showed good sensitivity close to the lower end of physiological range and required a shorter detection time than the other assays based on aptamers.^28^^,^^29^

### 3.3 Selectivity of the Assay

**Fig. 8:**
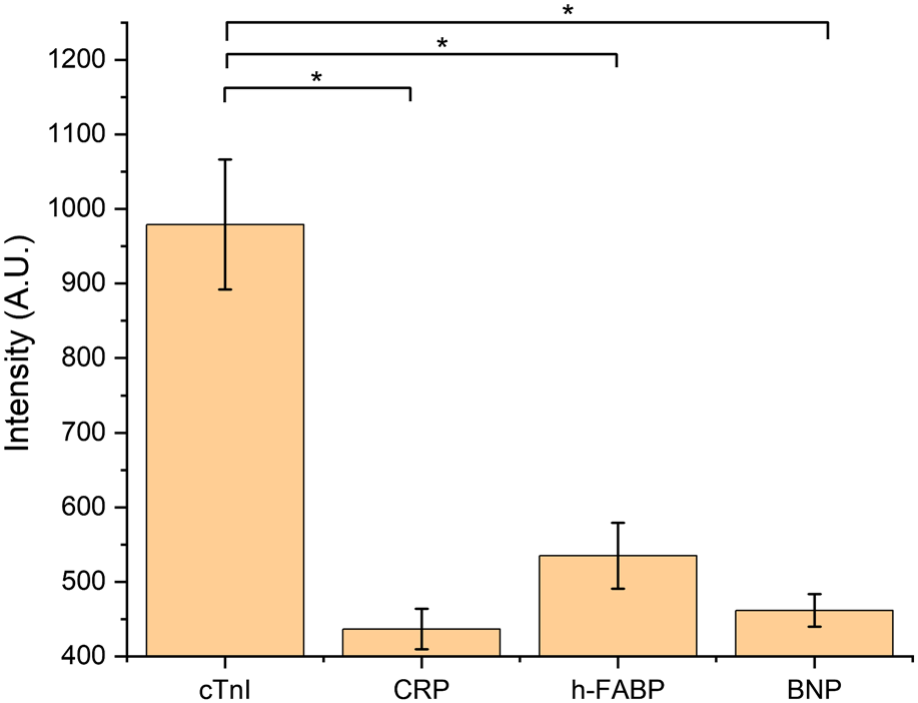
SERRS intensity at $1617\;{\mathrm{cm}}^{-1}$ of cTnI, CRP, h-FABP, and BNP, demonstrating the selectivity of the aptamer-based paper strip. Asterisks denote significantly different (p<0.05).

To evaluate the selectivity of the developed aptamer-based paper strip, potential interferents were tested all at the mid-level concentration of 0.1  ng/ml. CRP is an inflammatory biomarker and is used in risk stratification in cardiac diseases.^45^ The h-FABP is a protein and its concentration would increase after MI.^46^ BNP is a neurohormone released from the cardiac cells, and the increase in its concentration would indicate heart failure.^46^ Thus, CRP, h-FABP, and BNP were used to show the specificity to these potential interferents. These interferents were tested under the same experimental conditions as cTnI. Tests for each sample were performed in triplicate. Figure 8 shows the SERRS intensity at peak $1617\;{\mathrm{cm}}^{-1}$ measured from test lines of these samples. It is shown that the aptamer-based paper strip had low response to CRP and BNP. The signal intensity of h-FABP was higher than CRP and BNP, but as shown in Fig. 8, all three signals were statistically lower than cTnI. All three components were in the same range as the background (i.e., zero concentration of cTnI), indicating these interferents had a negligible change on the spectra from nonspecific binding. These results indicate the aptamer-based paper strip had a good selectivity to cTnI.

### 3.4 Stability of the Assay

**Fig. 9:**
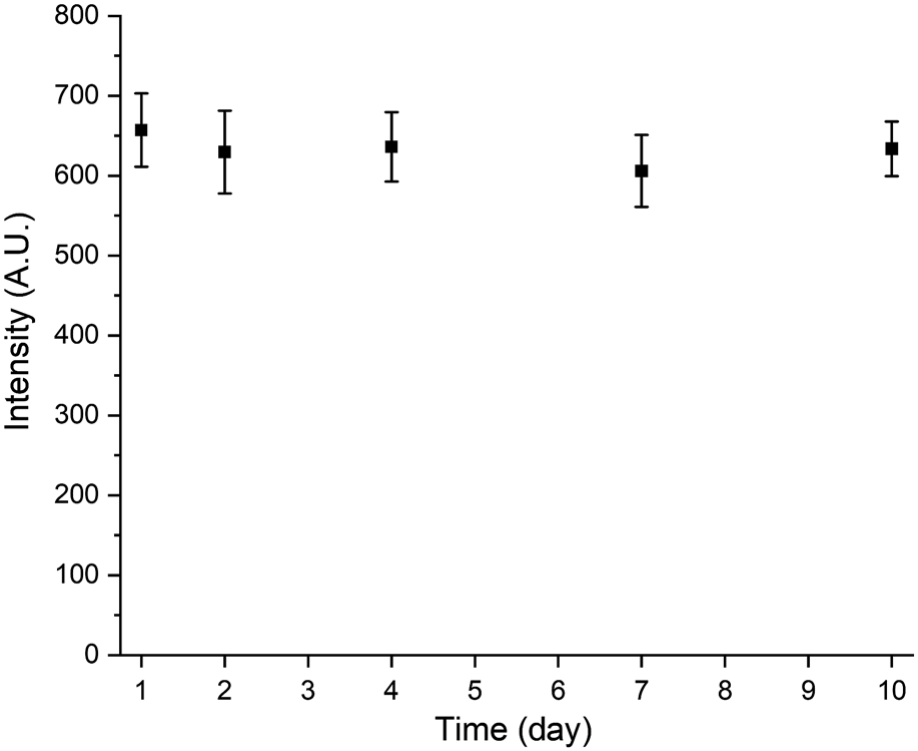
Stability of the aptamer-based paper strip stored at ambient conditions over 10 days.

To evaluate the stability of developed aptamer-based paper strip, the paper strip was stored in a sealed container at room temperature and its response to 0.03-ng/ml cTnI over 10 days was tested. As shown in Fig. 9, the SERRS signal changes over 10 days were within 8% of the initial response on day 1. On day 10, the signal retained 96.5% of the initial response on day 1. These results indicate that the developed aptamer-based paper strip had reasonable stability when stored at room temperature over 10 days.

### 3.5 Analysis of cTnI in Serum

To evaluate the performance of the aptamer-based paper strip for detection of cTnI in a complex biological sample matrix, a standard addition method was used to detect the recoveries of different concentrations of cTnI in human serum. The result is shown in Table 1. It can be observed that the recovery rate was in the range of 93.8% to 95.8% indicating the serum matrix had a low effect on the assay. No dilution of serum sample was needed in this assay due to the use of 50-μl running buffer, which helped the serum to flow and reduced the effect of higher viscosity of the serum. Therefore, the developed aptamer-based paper strip was shown to have the potential to be applied for the clinical determination of cTnI in a more complex medium.

**Table 1:** Test for the detection of cTnI in serum samples (n=3).

Sample	Added (ng/ml)	Found (ng/ml)	Recovery (%)	RSD (n=3) (%)
1	0.03	0.0285	93.83	4.99
2	0.05	0.0479	95.83	3.52

## 4 Conclusion

In this work, an aptamer-based SERRS assay on a paper strip for detection of cTnI was developed and characterized. The developed assay used aptamer rather than antibodies as the recognition element, due to its low relative cost, good thermal stability, tolerance to a range of pH levels, and long shelf life. The assay used silica/MGITC/AuNPs to provide a strong and stable SERRS signal at a 638-nm excitation wavelength. A handheld Raman spectrometer was used to measure the SERRS signal. A lateral flow strip design was used to build the paper fluidic strip. The particles were stored in the paper strip and ensured a simple operation for end-users. After introducing the sample with cTnI in the sample pad, a sandwich binding of the aptamer/silica/MGITC/AuNPs, the cTnI, and the aptamer on test line formed when cTnI was present. The SERRS signal on the test line increased with the concentration of cTnI. The developed assay for cTnI had a detection range of 0.016 to 0.1  ng/ml, with a LOD of 0.016  ng/ml. The developed assay also showed good selectivity to cTnI compared with other potential interferents. In addition, the developed assay showed stability over 10 days and good performance in spiked serum samples. To increase the sensitivity in the low-concentration range, a larger sample volume or a particle that produces a higher SERRS signal (e.g., nanostars and nanoshells) could be used in future tests. In addition, the assay time could be decreased using a nitrocellulose paper with a faster flow rate. Overall, this work showed that the aptamer-based SERRS assay on a paper platform has the potential to provide a sensitive, selective, stable, repeatable, and cost-effective platform for the detection of cTnI, toward eventual use in diagnosis of MI at the POC.

## Code, Data, and Materials Availability

The data that support the plots within this paper and other findings of this study are available from the corresponding author upon reasonable request.
