# Supplementary material for: Aptamer-based surface-enhanced resonance Raman scattering assay on a paper fluidic platform for detection of cardiac troponin I
Source: J Biomed Opt. 2020 Sep 8;25(9):097001. doi: 10.1117/1.JBO.25.9.097001 (PMC7477632; doi:10.1117/1.JBO.25.9.097001)
Supplement: Supplementary file 1 [file JBO_025_097001_SD001.docx]

**Supplementary materials**

Aptamer-based surface enhanced resonance Raman scattering assay on a paper fluidic platform for detection of cardiac troponin I

Dandan Tu ^*a^, Allison Holderby ^b^, Gerard L. Coté ^a,c^

a Texas A&M University, Department of Biomedical Engineering, College Station, Texas, United States

b Texas A&M University, Department of Chemistry, College Station, Texas, United States

c Texas A&M Engineering Experiment Station Center for Remote Health Technologies and Systems, College Station, Texas, United States

- **Supplementary data**

To evaluate the distribution of the particles on a test line on the paper strip, the following process was implemented. The SERS intensity on the test line area was measured using the mapping function in the Thermo Scientific DXR Raman confocal microscope with a 780 nm laser. A 10 × 40 point array with a 100 μm step size was measured on the test line area. Each point was measured by exciting with a 24 mW laser using a 3 s exposure. All spectra were baseline corrected. By using the peak intensity at around 1617 cm^-1^, the SERS intensity map could be drawn. **Figure S1** shows the SERS intensity map of the test line on a paper strip after testing 0.03 ng/mL of cTnI. It could be observed that the SERS signal at some spots are stronger. To overcome this variation, our work used a portable Raman spectrometer that has a Raster mode, which can scan a larger area on the test line than a single spot Raman measurement system. The Raman spectrometer also had a spot size of around 2.5 mm in its Raster mode, and hence it covered the thickness of the test line. An averaged intensity at 3 different spots of the test line from the left side to the right side was also used. Because the strip had a width of 4 mm and the spectrometer had a spot size of around 2.5 mm, three measurements was enough to cover the whole test line area. The final SERRS signal of the test line on a strip was the average of the intensity from these spectra.


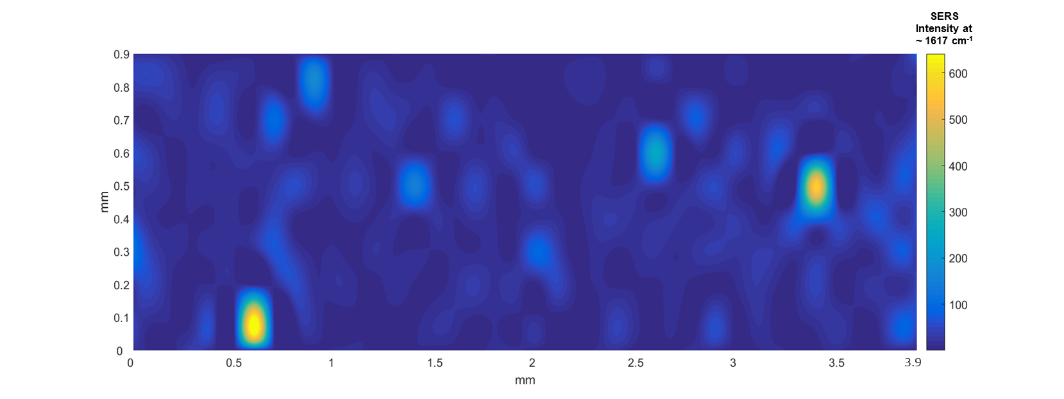


**Figure S1** SERS intensity distribution at the test line when testing 0.03 ng/mL of cTnI.

- **Comparison of the developed assay with previously reported assays for cTnI detection**

**Table S1.** Comparison of the developed assay with previously reported assays for cTnI detection

| No. | Transduction Mode | Portable Reader? (Y/N) | Recognition Element | Paper Based Platform? (Y/N) | Limit of Detection (ng/mL) | Detection Time (min) | Ref. |
| --- | --- | --- | --- | --- | --- | --- | --- |
| 1 | Electrochemiluminescence (ECL) | N | antibody | N | 0.0001 | 120 | (14) |
| 2 | Electrochemiluminescence (ECL) | N | antibody | N | 0.0032 | 240 | (24) |
| 3 | Fluorescence | N | antibody | Y | 0.049 | 15 | (25) |
| 4 | Electrochemistry | N | antibody | Y | 0.05 | 1 | (26) |
| 5 | Fluorescence | Y | antibody | Y | 0.01 | 10 | (16) |
| 6 | Electrochemistry | N | peptide | N | 0.0009 | 50 | (27) |
| 7 | Photoelectrochemical | N | aptamer | Y | 0.00000047 | 240 | (28) |
| 8 | Electrochemistry | N | aptamer | N | 0.0057 | 120 | (29) |
| 9 | Electrochemistry | Y | antibody | N | 0.02 | <10 | * (47) |
| 10 | - | Y | antibody | N | 0.018 | <10 | * (48) |
| 11 | Surface Enhanced Raman Spectroscopy (SERS) | Y | aptamer | Y | 0.016 | 40 | This work |

* Commercially available product
